# Phylogenetic inference of *Coxiella burnetii* by 16S rRNA gene sequencing

**DOI:** 10.1371/journal.pone.0189910

**Published:** 2017-12-29

**Authors:** Heather P. McLaughlin, Blake Cherney, Janetta R. Hakovirta, Rachael A. Priestley, Andrew Conley, Andrew Carter, David Hodge, Segaran P. Pillai, Linda M. Weigel, Gilbert J. Kersh, David Sue

**Affiliations:** 1 Laboratory Preparedness and Response Branch, National Center for Emerging and Zoonotic Infectious Diseases, Centers for Disease Control and Prevention, Atlanta, GA, United States of America; 2 Rickettsial Zoonoses Branch, National Center for Emerging and Zoonotic Infectious Diseases, Centers for Disease Control and Prevention, Atlanta, GA, United States of America; 3 Science and Technology Directorate, U.S. Department of Homeland Security, Washington, D.C., United States of America; 4 Office of Laboratory Science and Safety, Office of the Commissioner, U.S. Food and Drug Administration, Silver Spring, MD, United States of America; Johns Hopkins University, UNITED STATES

## Abstract

*Coxiella burnetii* is a human pathogen that causes the serious zoonotic disease Q fever. It is ubiquitous in the environment and due to its wide host range, long-range dispersal potential and classification as a bioterrorism agent, this microorganism is considered an HHS Select Agent. In the event of an outbreak or intentional release, laboratory strain typing methods can contribute to epidemiological investigations, law enforcement investigation and the public health response by providing critical information about the relatedness between *C*. *burnetii* isolates collected from different sources. Laboratory cultivation of *C*. *burnetii* is both time-consuming and challenging. Availability of strain collections is often limited and while several strain typing methods have been described over the years, a true gold-standard method is still elusive. Building upon epidemiological knowledge from limited, historical strain collections and typing data is essential to more accurately infer *C*. *burnetii* phylogeny. Harmonization of auspicious high-resolution laboratory typing techniques is critical to support epidemiological and law enforcement investigation. The single nucleotide polymorphism (SNP) -based genotyping approach offers simplicity, rapidity and robustness. Herein, we demonstrate SNPs identified within 16S rRNA gene sequences can differentiate *C*. *burnetii* strains. Using this method, 55 isolates were assigned to six groups based on six polymorphisms. These 16S rRNA SNP-based genotyping results were largely congruent with those obtained by analyzing restriction-endonuclease (RE)-digested DNA separated by SDS-PAGE and by the high-resolution approach based on SNPs within multispacer sequence typing (MST) loci. The SNPs identified within the 16S rRNA gene can be used as targets for the development of additional SNP-based genotyping assays for *C*. *burnetii*.

## Introduction

*Coxiella burnetii* is the etiological agent of Q fever, a zoonotic disease which can result in large outbreaks because of its low infectious dose. The main route of transmission to humans is inhalation of contaminated aerosols which are capable of traveling on wind currents for miles [1]. Although less common, outbreaks have been reported following ingestion of unpasteurized milk and dairy products as well as through contact with contaminated clothing [2, 3]. Clinical manifestations of the acute form can range from flu-like symptoms to pneumonia and in some cases result in death caused by respiratory distress [4]. As the acute form presents with non-specific symptoms, healthcare providers are often challenged with accurate diagnosis for Q fever. Centers for Disease Control and Prevention (CDC) recommends a combination of serology testing complemented with polymerase chain reaction (PCR) of clinical specimens for definitive laboratory diagnosis of early stage acute Q fever [5]. Persistent focalized *C*. *burnetii* infections, the chronic form, can present as endocarditis [4, 6–8], vascular or osteoarticular infections [9, 10] and are primarily diagnosed by serological testing. The low infectious dose (less than 10 bacteria), high resistance to environmental stress in its nonreplicating small-cell variant (SCV) form, and potential for deliberate dissemination are the basis for classifying *C*. *burnetii* as a select agent regulated by the Federal Select Agent Program (https://www.selectagents.gov/SelectAgentsandToxinsList.html) [11]. This highly virulent pathogen has been weaponized by both the U.S. and former U.S.S.R. under various biological warfare programs [12] and is considered a bioterrorism concern for both civilians and military personnel.

The epidemiology of Q fever is complex due to its world-wide geographic distribution and diverse reservoirs that include wild and domestic animals, livestock, arthropods, and humans [4]. Epidemics of this zoonosis have been reported in residential areas as a result of dissemination from nearby farms [13], among troops during wartime [14–16], and can be hyperendemic in certain regions with sporadic cases spanning several decades [17]. The largest reported global outbreak occurred in the Netherlands between 2007 and 2010 [18]. Despite the high prevalence of *C*. *burnetii* in the environment, epidemiological data are limited because isolate collections are usually small and are governed by select agent regulations [19]. The advent of host cell-free anexic media and the contribution of genomics were major advances in the study of Q fever. Whole genome sequencing (WGS) of *C*. *burnetii* has revealed genetic diversity between strains and has enabled the development of new molecular typing methods that will help bridge gaps in epidemiological knowledge and define phylogenic relatedness. It has been demonstrated that the epidemiological features and clinical presentation of acute Q fever can be strain dependent [20]. Simple and rapid laboratory genotyping methods would aid in trace-back investigations during natural or intentional outbreaks or other public health events.

Numerous molecular typing methods for *C*. *burnetii* have been described in the literature and applied to epidemiological investigations involving naturally occurring outbreaks and a suspected intentional release [21]. Initial typing methods used before 2005 were based on plasmid types [22], pulsed-field gel electrophoresis (PFGE) [23], restriction fragment length polymorphisms (RFLP) analyzed with SDS-PAGE [24], and sequence analysis of genes including *com1*, *mucZ*, *icd* and *16S/23S* [25–28]. These methods were shown to have varying degrees of discriminatory power and their routine use could not be established as a result of limited reproducibility within and between laboratories [21]. Recently, more high resolution sequence-based genotyping methods such as multispacer sequence typing (MST) [29] and multiple locus variable number of tandem repeats (VNTR) analysis (MLVA) [30] have become the most commonly used approaches. Although the new genotyping methods have increased discriminatory power between *C*. *burnetii* strains, converting to these new methods frequently reduces the amount of information originally obtained by more traditional methods, as data may be discarded or cannot be directly compared [19]. According to Hornstra *et al*. [19], new typing methods should be unambiguous, simply transferrable and allow for *C*. *burnetii* typing results to be compared to existing collections. In 2011, Hornstra and colleagues used the publicly available MST scheme from the largest and most diverse *C*. *burnetii* collection in the world [29] to design SNP assays that could be used to type isolates based on previously described phylogenetic groups.

Phylogenetic analysis, mainly based on 16S ribosomal RNA (rRNA) gene sequences, has placed *Coxiella* in the Class *Gammaproteobacteria* along with *Legionella*, *Francisella*, and *Rickettsiella* [27, 31]. It has also assigned all *Coxiella* isolates to the single *C*. *burnetii* species [27, 32]. While 16S rRNA gene sequencing has confirmed the phylogenic homogeneity of *C*. *burnetii* strains, reports suggest this method does not have sufficient discriminatory power to be used for genotyping [27]. It has also been demonstrated that other classical genotyping methods based on 16S-23S internal transcribed spacer sequencing and RNA polymerase’s β-subunit (*rpoB*) sequencing are not useful for epidemiological studies due to high levels of sequence homology [28, 33]. Here, we demonstrate 16S rRNA sequencing does indeed differentiate *C*. *burnetii* strains and can be used as a simple, unambiguous, transferrable genotyping approach. To maintain synchronization between typing methods, strains used in this study that overlapped with strains genotyped by other methods were directly compared.

## Materials and methods

### Bacterial strains and DNA extraction

*C*. *burnetii* strains included in this study and respective epidemiological information are listed in **Table 1**. To synchronize results between typing methods we compared 16 strains that overlapped with the Hendrix *et al*. [24] study and 39 strains that overlapped with the Hornstra *et al*. [19] study. Genomic DNA was isolated using the QIAamp DNA Mini Kit (Qiagen, Valencia, CA) following the manufacturer’s “DNA Purification from Tissues” protocol with overnight proteinase K lysis at 56°C.

**Table 1:** 55 *Coxiella burnetii* strains used in this study and SNPs within their respective 16S rRNA genes.

					16S rRNA gene nucleotide positions				
Strain	Alternate Names	Original Source	Location	Year	64	164	622	830	1022
**Nine Mile (Ref)**	RSA 493, phase I, 9Mi	Tick	Montana, USA	1935	G	C	C	A	C
Ohio	RSA 270	Cow milk	Ohio, USA	1958	G	C	C	A	C
Turkey	RSA 315	Human blood	—	1948	G	C	C	A	C
O. megnini	—	Tick (*Otobius megnini*)	—	1948	G	C	C	A	C
Cypriot	—	—	—	—	G	C	C	A	C
Scottish	—	—	Scotland	—	G	C	C	A	C
I. scapularis	—	Tick (*Ixodes scapularis*)	—	—	G	C	C	A	C
El Tayeb	RSA 342	Tick	Egypt	1967	G	C	C	A	C
B1 Cyprus Ovine	—	Sheep	Cyprus	—	G	C	C	A	C
CM-CA1	—	Cow milk	California, USA	2007	G	C	C	A	C
Dyer	RSA 345	Human blood	Montana, USA	1938	G	C	C	A	C
ES-VA1	—	Environment	Virginia, USA	2010	G	C	C	A	C
R. sanguineus	—	Tick (*Rhipicephalus sanguineus*)	—	1949	G	C	C	A	C
Nine Mile Crazy	RSA 514	Tick	Montana, USA	1935	G	C	C	A	C
CM-SC1	—	Cow milk	S. Carolina, USA	2007	G	C	C	A	C
Panama	RSA 335	Chigger and mite pool	Panama	1961	G	C	C	A	C
D. occidentalis	—	Tick (*Dermacentor occidentales*)	—	1940	G	C	C	A	C
Dugway 5G61-63	—	Tick *(Dermacentor parumaperlus*)	Utah, USA	1958	G	C	C	A	C
Q155	—	—	—	—	G	C	C	A	C
Giroud Banqui Q	RSA 431	Human blood	Central Africa	1949	G	C	C	A	C
Dugway 7D77-80	—	Rodent	Utah, USA	1957	G	C	C	A	C
Australian QD	RSA 425	Human blood	Australia	1939	G	C	C	A	C
A. americanum	—	Tick (*Amblyomma americanum*)	—	—	G	C	C	A	C
Kmen L35	—	—	—	—	G	C	C	A	C
CS 27	Kmen 27	Tick	Slovak Republic	1967	G	C	T	A	C
M44	RSA 459, Grita	Human blood	Italy	1945	G	C	T	A	C
Henzerling	RSA 331	Human blood	Northern Italy	1945	G	C	T	A	C
Paige	—	Human, acute	Italy	1946	G	C	T	A	C
Florian	—	Human blood	Slovak Republic	1956	G	C	T	A	C
CS S1	Kmen S1	Cow	Russia	—	G	C	T	A	C
Cb 48	—	Tick *(Haemophysalis punctate)*	Slovak Republic	1967	G	C	T	A	C
Arandale	—	Human blood	Australia	—	A	C	C	C	C
Resson	—	—	—	—	A	C	C	C	C
HHV-WA1	—	Human heart valve	Washington, USA	2007	A	C	C	C	C
K	Q154, KAV	Human heart valve	Oregon, USA	1979	A	T	C	C	C
GP-MT2	—	Goat Placenta	Montana, USA	2011	A	T	C	C	C
ES-WA1	—	Environmental Sample	Washington, USA	2011	A	T	C	C	C
GP-WA1	—	Goat Placenta	Washington, USA	2011	A	T	C	C	C
P	Q238, Q173, PAV	Human heart valve	California, USA	1979	A	T	C	C	C
Priscilla	Q177, MSU goat	Goat cotyledon	Montana, USA	1980	A	T	C	C	C
ES-MT1	—	Environmental Sample	Montana, USA	2012	A	T	C	C	C
GP-AF1	—	Goat Placenta	Afghanistan	2011	A	T	C	C	C
ES-WA2	—	Environmental Sample	Washington, USA	2012	A	T	C	C	C
Canada Goat	Q218	Goat	Canada	1981	A	T	C	C	C
ES-CA1	—	Environment	California, USA	2011	A	T	C	C	C
HHV-WA2	—	Human heart valve	Washington, USA	2010	A	T	C	C	C
GS-MT1	—	Goat vaginal swab	Montana, USA	2011	A	T	C	C	C
W	WAV	Human heart valve	—	—	G	C	C	A	T
S	Q217	Human liver biopsy	Montana, USA	1981	G	C	C	A	T
G	Q212	Human heart valve	Nova Scotia	1981	G	C	C	A	T
L	Q216	Human heart valve	Nova Scotia	1981	G	C	C	A	T
Ko	Q229	Human heart valve	Nova Scotia	1982	G	C	C	A	T
Poker Cat	—	Cat uterus	Nova Scotia	1986	G	C	C	A	T
McMaster	Q217	Human placenta	—	—	G	C	C	A	T
Mauriet	—	Human	France	—	G	C (A+) C		A	C

Nucleotide positions within the 16s rRNA gene correspond to sequence alignment with the Nine Mile reference (Ref) strain. A plus sign (+) indicates the insertion of adenine at position 213.

### 16S rRNA gene amplification and Sanger sequencing

16S rRNA genes from 55 *C*. *burnetii* strains were amplified by PCR using the universal, bacteria-specific primers E8F and E1541R to obtain an amplicon of approximately 1500 bp [34]. PCR amplification reactions were performed as outlined in Hakovirta *et al*. [35] and products were confirmed by gel electrophoresis using a 0.8% E-Gel® electrophoresis system (Life Technologies, Eugene, OR). ExoSAP-IT (ThermoFisher Scientific, Pittsburgh, PA) was used to hydrolyze any remaining PCR primers in the amplification reactions and DNA cycle sequencing reactions were performed with the Big Dye® Terminator v 3.1 cycle sequencing kit (ThermoFisher Scientific, Pittsburgh, PA). 16S rRNA gene sequences were generated using five previously described, bi-directional oligonucleotide primers [34, 35] and two additional primers, E341R (TGCIGCCICCCGTAGG) and E1115F (CAACGAGCGCAACCCT), designed to increase sequencing coverage at the 3’ and 5’ ends. Reaction products generated from cycle sequencing were purified using the BigDye® XTerminator Purification kit (Life Technologies, Eugene, OR) and DNA was sequenced using an Applied Biosystems 3500xL Genetic Analyzer and KB Basecaller Software v1.4.1 (Life Technologies, Eugene, OR).

### DNA sequence analysis and identification of 16S rRNA SNPs

16S rRNA contig assemblies, alignments, and identification of SNPs were performed with Sequencher™ v5.0 software (Gene Codes Corporation, Ann Arbor, MI). Prior to assembling a 16S rRNA consensus sequence, the electropherogram for each DNA sequence was visually examined and trimmed for quality. Fifty-five 16S rRNA consensus sequences generated in this study were compared with other 16S rRNA gene sequences in Ribosomal Database Project (RDP, http://rdp.cme.msu.edu) and in GenBank of NCBI (https://blast.ncbi.nlm.nih.gov) using the Basic local alignment search tool (BLAST) [36] to confirm the *C*. *burnetii* species. Gene sequences for all strains generated herein, along with two additional sequences obtained from NCBI for *C*. *burnetii* Z3055 (Accession number NZ_LK937696) and 3262 (Accession number CP013667), were then aligned to the 16S rRNA reference sequence of *C*. *burnetii* Nine Mile. Position numbers for the five SNPs identified in this study were assigned based on alignment to this reference sequence. A discriminatory power calculator (http://insilico.ehu.es/mini_tools/discriminatory_power/index.php) was used to calculate discriminatory power (D). D is a single numerical index of discrimination expressed by the formula of Simpson’s index of diversity and is based on the probability that two unrelated strains sampled from the test population will be placed in different typing groups [37]. These values were calculated based on 36 strains and 6 types for the Hendrix *et al*. [24] study, 50 strains and 10 types for the Hornstra *et al*. [19] study and 50 strains and 6 types for this study. As epidemiological data is limited for many of the historical *C*. *burnetii* isolates, relatedness could not be established between all strains. In our study, the six known epidemiologically related strains, GP-MT2, ES-WA1, GP-WA1, ES-MT1, ES-WA2 and GS-MT1, were considered as one for the purpose of calculating D.

### 14 SNP-based genotyping assay

Hornstra *et al*. [19] identified polymorphisms within MST loci and designed a genotyping assay based on 14 SNPs. SNP data for 39 of the 55 *C*. *burnetii* strains examined in this work have previously been published [19]. SNP assay results for the remaining 16 strains were obtained as part of this work. Briefly, 12 SNPs were used to develop Melt-Mismatch Amplification Mutation Assays (MAMA), and melt curves were analyzed according to Vogler *et al*. [38]. Two SNPs were used to develop TaqMan minor groove binding dual-probe assays, and results were analyzed as described by Easterday *et al*. [39]. For all 14 assays, 1 μL of DNA was used in a total reaction volume of 10 μL. 1 x SYBR Green PCR Master Mix and 1 x TaqMan Genotyping Master Mix (both by Life Technologies, Foster City, CA) were used for the Melt-MAMA assays and TaqMan minor groove-binding dual-probe assays, respectively. Thermal cycling conditions, allele-specific primers, instruments and software details are all outlined in Hornstra *et al*. [19].

### Phylogenetic tree assembly

16S rRNA gene sequences were aligned using the multiple sequence alignment method MUSCLE and overhanging ends were trimmed in the software suite MEGA v6.06. Pairwise distances between all *C*. *burnetii* strains were computed for both the 16S rRNA and genomic SNP sites, and neighbor-joining was used to infer phylogeny from the observed pairwise distances. Stability of the 16S rRNA tree was tested by bootstrapping 500 times.

## Results

### Identification of 16S rRNA gene single nucleotide polymorphisms

**Fig 1:**
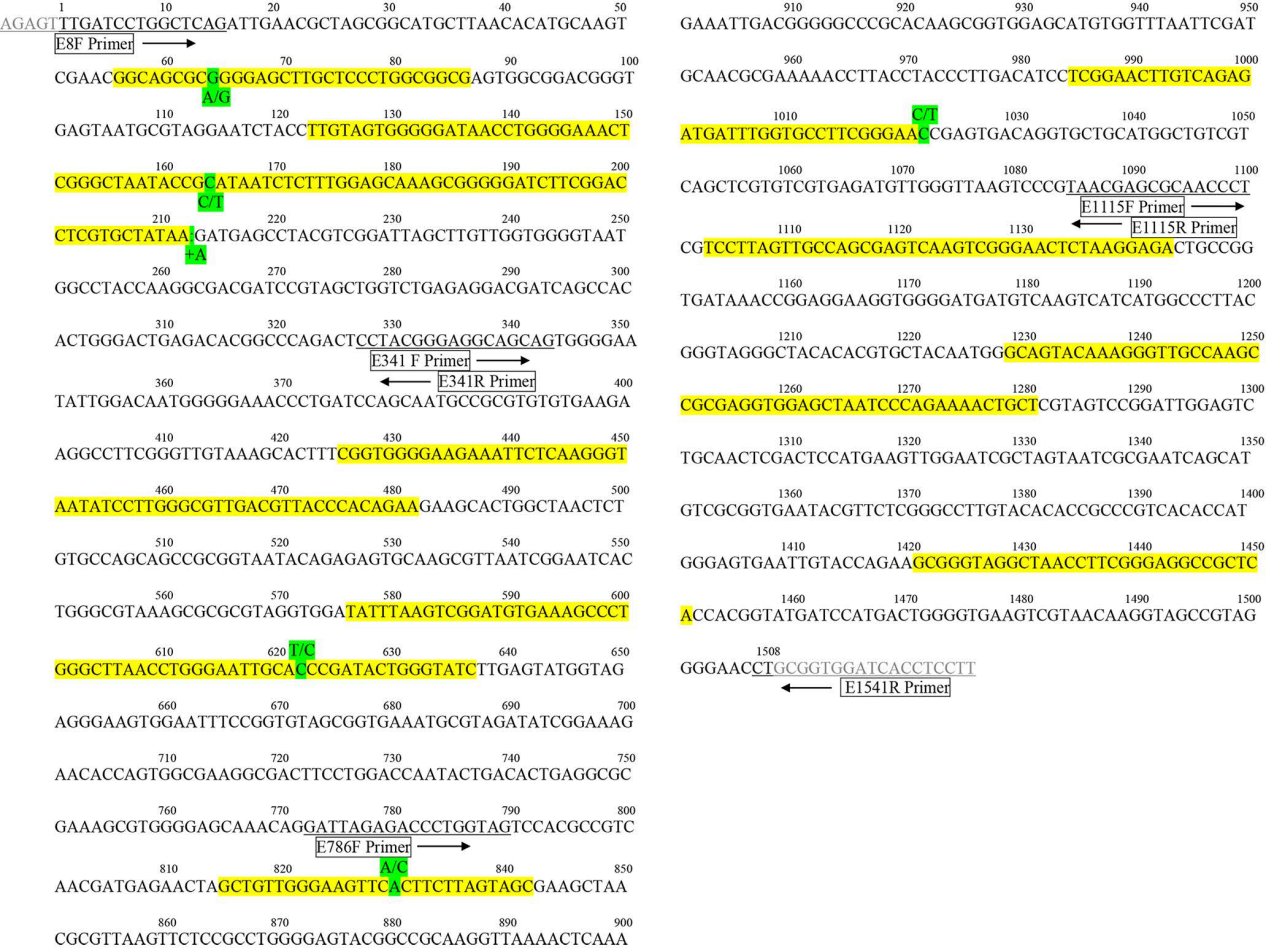
16S rRNA gene sequence of the *C*. *burnetii* Nine Mile reference strain. Five SNPs and one insertion polymorphism identified among the 55 *C*. *burnetii* 16S rRNA gene sequences are highlighted in green. The nine variable regions corresponding to those reported in the variability map of the *E*.*coli* 16S rRNA gene [34, 40] are highlighted in yellow. Bi-directional primers used to generate 16S rRNA sequences are boxed and priming sites are underlined.

*C*. *burnetii* strains included in this study and the SNPs within their respective 16S rRNA genes are listed in **Table 1**. A diverse set of 55 *C*. *burnetii* strains isolated from various geographical locations and host reservoirs were chosen for analysis in this study. 16S rRNA gene sequences from these strains were determined for amplified product with lengths greater than or equal to 1500 nucleotides. When sequences were aligned and analyzed, six polymorphisms were identified among the 55 strains; five SNPs and one nucleotide insertion. Nucleotide position numbers for these six polymorphisms were assigned based on sequence alignment to the *C*. *burnetii* Nine Mile reference strain and their locations are illustrated in green in **Fig 1**. These six positions are all located within or directly flanking one of nine determined variable regions based on a variability map produced for the *Escherichia coli* 16S rRNA gene [34, 40]. The nine regions within the 16S rRNA gene of the *C*. *burnetii* Nine Mile reference strain corresponding to the variable regions reported in *E*.*coli* are highlighted in yellow (**Fig 1**). Three of the five SNPs identified at nucleotide positions 164, 622 and 1022 are pyrimidine transitions with either a thymine or cytosine present in all strains. The SNP located at position 64 represents a purine transition where a guanine was confirmed in 39 of the 55 strains and an adenine in the remaining 16. A transversion mutation involving an adenine/cytosine exchange represents the SNP at position 820.

**Fig 2:**
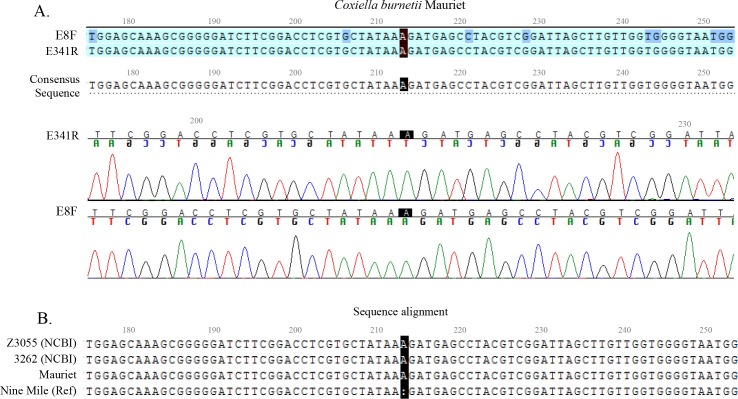
Insertion polymorphism identified in the 16S rRNA gene of *C*. *burnetii* Mauriet. (A) DNA sequences obtained using primers E8F and E341R designate an adenine insertion at nucleotide position 213 which is confirmed in the electropherograms generated by Sequencher™ analysis software. (B) Sequence alignment shows 16S rRNA genes of *C*. *burnetii* Z3055 (GenBank accession number NZ_LK937696) and 3262 (GenBank accession number CP013667) also possess this insertion which is absent in the Nine Mile reference (Ref) strain sequence.

*C*. *burnetii* Mauriet was the only strain in our study in which an insertion was identified at nucleotide position 213 (**Fig 2**). This indel was detected on DNA sequences obtained using both the forward and reverse sequence that cover this region. Visual inspection of the two electropherograms generated from these DNA sequences also confirmed this insertion (**Fig 2A**). The 16S rRNA sequence of the Mauriet strain was then compared to those found in the GenBank database of NCBI using BLAST. Two *C*. *burnetii* strains, Z3055 (accession number NZ_LK937696) and 3262 (accession number CP013667), were shown to possess an A inserted at position 213 which is absent in the Nine Mile reference strain sequence (**Fig 2B**). Although not detected using the BLAST search, this indel was also identified in the 16S rRNA gene sequence of *C*. *burnetii* Cb109 (data not shown).

### Genotyping by 16S rRNA SNP analysis

**Fig 3:**
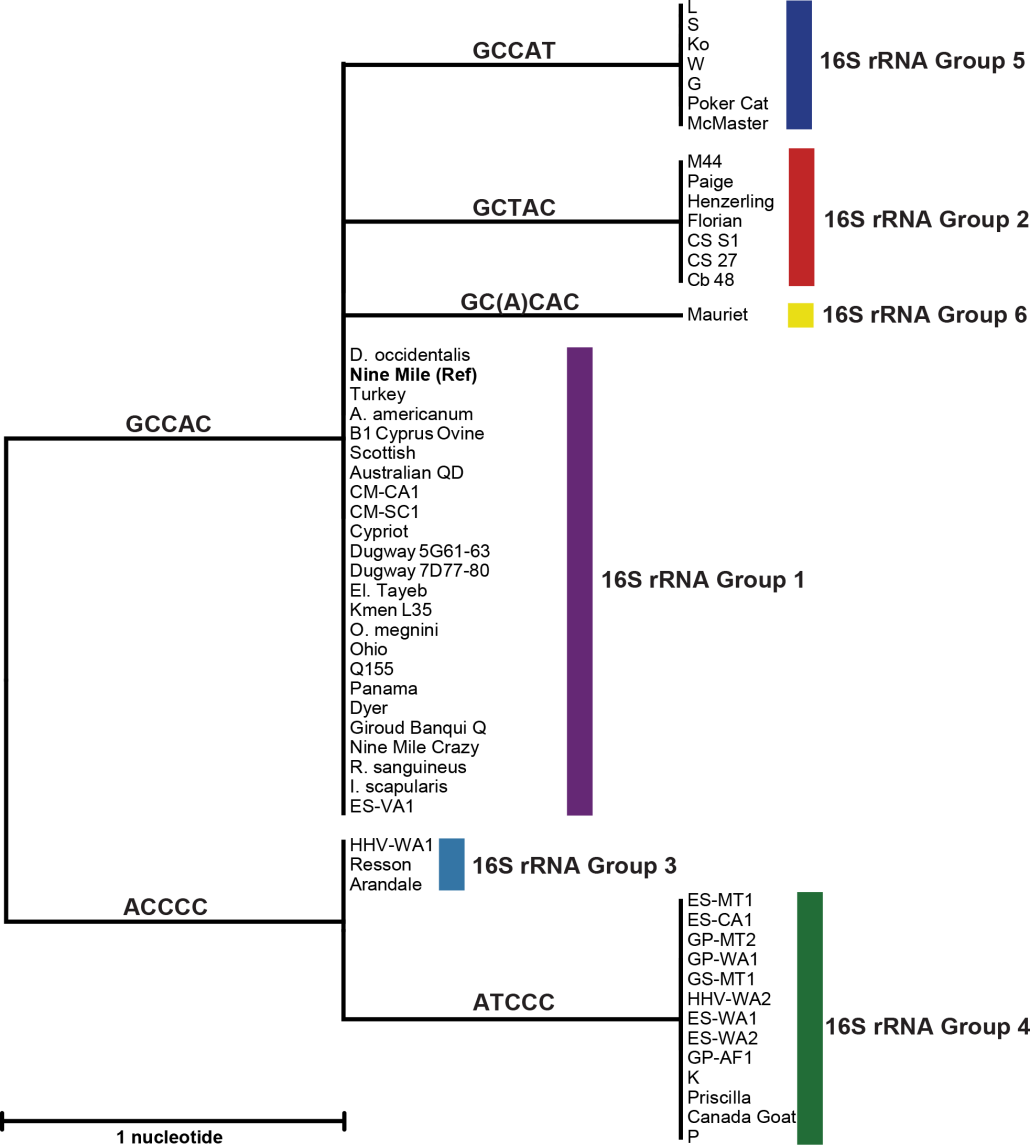
16S rRNA gene SNP-based phylogeny of *C*. *burnetii*. This rooted phylogenetic tree was created from pairwise distances observed from 16S rRNA gene SNP sites. The five SNPs and one insertion identified among the 55 16S rRNA gene sequences gave rise to six groups (color coded). Based on sequence alignment to the 16S rRNA gene of *C*. *burnetii* Nine Mile (bold), SNPs at positions 64, 164, 622, 830, and 1022 and an insertion at positon 213 (in parentheses) gave rise to the six distinct SNP signatures, which are displayed on their respective branches.

The neighbor-joining clustering method was used to create a phylogenetic tree from pairwise distances observed from SNP sites between all *C*. *burnetii* strains (**Fig 3**). The five SNPs and one indel identified among the 55 16S rRNA gene sequences gave rise to six genotypes. The largest number of isolates, including the Nine Mile reference strain, was assigned to Group 1 and possesses the SNP signature GCCAC at positions 64, 164, 622, 830, and 1022, respectively. Strains clustered in this group have worldwide distribution originating in North America, Africa, Central America, Europe, and Australia (**Table 1**). The 16S rRNA gene of *C*. *burnetii* Mauriet contains the same five SNPs as Group 1, and the indel at position 213 situates this strain separately in Group 6. The five SNPs in the 16S rRNA sequences of strains Z3055 and 3262 obtained from GenBank were determined to be identical to those confirmed in the Mauriet strain and would also place them in Group 6 (data not shown).

One SNP separates both Groups 2 and 5 from Group 1 where GCTAC is observed for Group 2 and GCCAT for Group 5. The geographic distributions of strains assigned to these two groups are more concentrated compared to Group 1. Group 2 includes 7 strains isolated in the mid-20^th^ century from Europe and Russia whereas strains in Group 5 originate from Nova Scotia and the northern United States, all of which were isolated in the 1980s (**Table 1**). Strains assigned to Group 3 form a separate cluster at the initial bifurcation point of the phylogenetic tree and contain the SNP signature ACCCC (**Fig 3**). Members of this Group are associated with Australia [41] and the HHV-WA1 strain was isolated from a person with travel history to that country and who was likely exposed there. Thirteen *C*. *burnetii* strains occupy Group 4 on the next closest branch and differ by only a T transition at position 164. The majority of strains associated with Group 4 were isolated from goats or environments from the north western part of the United States. The three human heart valve isolates in this group, K, P, and HHV-WA2, were also found in the same region of the United States located in Oregon, California, and Washington states, respectively.

### Typing method comparisons

To evaluate the discriminatory power of the 16S rRNA SNP-based genotyping method, we compared our inference of *C*. *burnetii* phylogeny to inferences obtained using other molecular typing methods. Sixteen isolates overlapping with those used in the Hendrix *et al*. [24] study, which defines genomic groups based on restriction enzyme banding patterns, were compared. In addition, 39 overlapping isolates included in the Hornstra *et al*. [19] study that used SNP signatures among MST loci were also assessed. These comparative results are outlined in **Table 2**along with observed MST genotypes reported by Hornstra *et al*. [19]. Based on DNA restriction fingerprints, Hendrix *et al*. [24] differentiated *C*. *burnetii* isolates into six distinct genomic groups which is comparable to the results obtained herein. The strains assigned to 16S rRNA Groups 3 and 6 were not represented in the RE/SDS-PAGE study and vice versa. All 16 strains in common from the remaining four groups fell within their corresponding genomic groups (**Table 2**). The discriminatory powers for these two typing schemes are similar with D values of 0.7151 and 0.7524 for the 16S rRNA gene-based method and the RE/SDS-PAGE method, respectively.

**Table 2:** Comparison of typing methods.

**16S rRNA Group 1** GCCAC	RE/SDS-PAGE Genomic Group Hendrix *et al*.	Predicted Genomic Group Hornstra *et al*. This work		Observed MST Genotype Hornstra *et al*. This work	
D. occidentalis	n/a	n/a	1	n/a	16,26
**Nine Mile (Ref)**	1	1	-	16,26	-
Turkey	n/a	1	-	16,26	-
A. americanum	n/a	n/a	1	n/a	16,26
B1 Cyprus Ovine	n/a	1	-	16,26	-
Scottish	n/a	1	-	16,26	-
Australian QD	1	1	-	16,26	-
CM-CA1	n/a	3	-	20	-
CM-SC1	n/a	3	-	20	-
Cypriot	n/a	1	-	16,26	-
Dugway 5G61-63	n/a	1	-	16,26	-
Dugway 7G77-80	n/a	n/a	6	n/a	Dugway
El. Tayeb	n/a	n/a	1	n/a	16,26
Kmen L35	n/a	1	-	16,26	-
O. mengnini	n/a	n/a	1	n/a	16,26
Ohio	1	1	-	16,26	-
Q155	n/a	1	-	16,26	-
Panama	n/a	n/a	1	n/a	16,26
Dyer	1	1	-	16,26	-
Giroud Banqui Q	1	1	-	16,26	-
Nine Mile Crazy	1	1	-	16,26	-
R. sanguineus	n/a	n/a	1	n/a	16,26
l. scapularis	n/a	n/a	1	n/a	16,26
ES-VA1	n/a	1	-	16,26	-
**16S rRNA Group 2** GCTAC	RE/SDS-PAGE Genomic Group Hendrix *et al*.	Predicted Genomic Group Hornstra *et al*. This work		Observed MST Genotype Hornstra *et al*. This work	
M44	2	2	-	18, 25	-
Paige	n/a	2	-	18, 25	-
Henzerling	2	2	-	18, 25	-
Florian	n/a	2	-	22, 23, 29	-
CS S1	n/a	2	-	22, 23, 29	-
CS 27	n/a	2	-	22, 23, 29	-
Cb 48	n/a	2	-	22, 23, 29	-
**16S rRNA Group 3** ACCCC	RE/SDS-PAGE Genomic Group Hendrix *et al)*	Predicted Genomic Group Hornstra *et al*. This work		Observed MST Genotype Hornstra *et al*. This work	
HHV-WA1	n/a	4	-	1–7, 30	-
Resson	n/a	4	-	1–7, 30	-
Arandale	n/a	4	-	1–7, 30	-
**16S rRNA Group 4** ATCCC	RE/SDS-PAGE Genomic Group Hendrix *et al*.	Predicted Genomic Group Hornstra *et al*. This work		Observed MST Genotype Hornstra *et al*. This work	
ES-MT1	n/a	n/a	4	n/a	8
ES-CA1	n/a	4	-	8	-
GP-MT2	n/a	n/a	4	n/a	8
GP-WA1	n/a	n/a	4	n/a	8
GS-MT1	n/a	n/a	4	n/a	8
HHV-WA2	n/a	4	-	8	-
ES-WA1	n/a	n/a	4	n/a	8
ES-WA2	n/a	n/a	4	n/a	8
GP-AF1	n/a	n/a	4	n/a	27,28,31
K	4	4	-	8	-
Priscilla	n/a	4	-	8	-
Canada Goat	n/a	n/a	4	n/a	8
P	4	4	-	8	-
**16S rRNA Group 5** GCCAT	RE/SDS-PAGE Genomic Group Hendrix *et al*.	Predicted Genomic Group Hornstra *et al*. This work		Observed MST Genotype Hornstra *et al*. This work	
L	5	5	-	21	-
S	5	5	-	21	-
Ko	5	5	-	21	-
W	n/a	5	-	21	-
G	5	5	-	21	-
Poker Cat	n/a	5	-	21	-
McMaster	n/a	5	-	21	-
**16S rRNA Group 6** GC(+A)CAC	RE/SDS-PAGE Genomic Group Hendrix *et al*.	Predicted Genomic Group Hornstra *et al*. This work		Observed MST Genotype Hornstra *et al*. This work	
Mauriet	n/a	2	-	11–15, 24, 32–34	-
Z3055 (NCBI)	n/a	n/a	-	n/a	-
3262 (NCBI)	n/a	n/a	-	n/a	-

Comparative genotyping results between the 16S rRNA gene-based method and two other typing methods. RE/SDS-PAGE genomic groups were described by Hendrix *et al*. [24]. Predicted genomic groups and observed MST genotypes were described by Hornstra *et al*. [19]. *C*. *burnetii* strains not included in the Hendrix and Hornstra studies are indicated as not available (n/a). The predicted genomic groups and observed MST genotypes performed herein for 16 of the 55 strains not included in the Hornstra study are listed as part of this work. Dashes (-) are used for the remaining 39 strains previously genotyped by Hornstra *et al*. [19].

**Fig 4:**
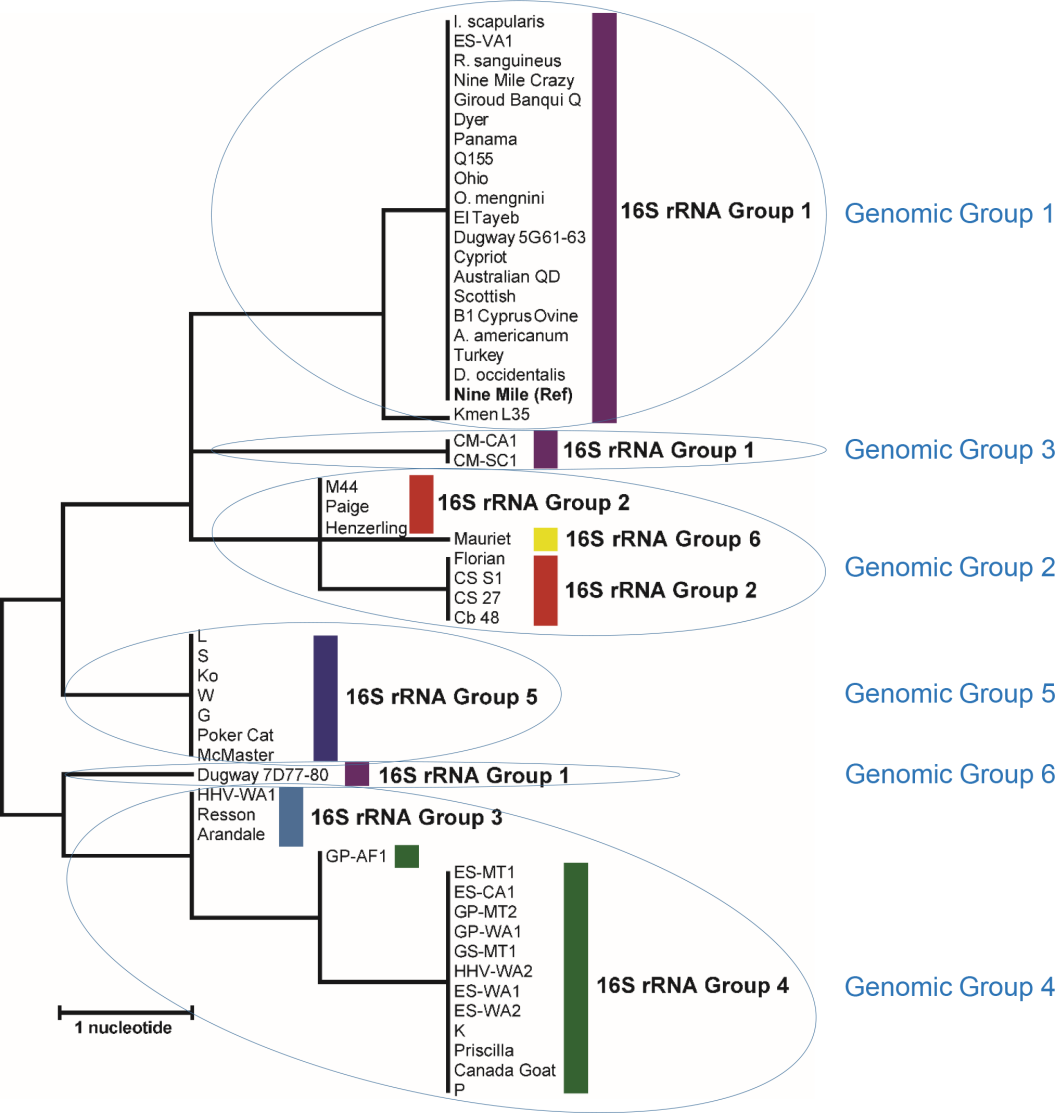
MST loci SNP-based phylogeny of *C*. *burnetii*. This rooted phylogenetic tree was created from pairwise distances observed from 14 SNP sites within MST loci. SNP positions in *C*. *burnetii* Nine Mile (bold) are outlined in Hornstra *et al*. [19]. The predicted genomic groups from this phylogenetic analysis are circled in blue and the 16S rRNA gene-based groups are color coded for comparison.

The genotyping assay based on 14 SNP sites described by Hornstra *et al*. [19] provides discrimination between the genomic groups reported by Hendrix *et al*. [24] with some resolution within these groups. While SNP signatures for 39 of the 55 study isolates have previously been reported, signatures for the remaining isolates were examined by Melt-MAMA or dual-probe assays as part of this work to increase the dataset for comparison (**S1 Table**). The predicted genomic groups and observed MST genotypes for these additional 16 strains can be found in **Table 2**. To illustrate phylogenetic inference of *C*. *burnetii* based on these SNP signatures, the neighbor-joining clustering method was also used to create a tree from pairwise distances observed from 14 SNP sites within MST loci (**Fig 4**). While 14 polymorphic sites led to increased resolution within this tree, all but two of the 55 strains were assigned to either the correct predicted genomic group or located on the nearest branch indicating the closest evolutionary relationship. This can be illustrated by the color-coded 16S rRNA Groups indicated in **Fig 4**. All the isolates that clustered into the 16S rRNA Groups 2 and 5 fell within their corresponding predicted genomic group. Twenty-two of the 24 isolates of 16S rRNA Group 1 also fell within the predicted genomic group 1 with observed MST genotypes of 16 and 26. While the two remaining 16S rRNA Group 1 isolates, CM-CA1 and CM-SC1, belong to a different MST genotype within predicted genomic group 3, they are the nearest neighbors on the tree. Similarly, while the 16S rRNA Group 4 isolate *C*. *burnetii* GP-AF1 populated an individual branch due to its unique MST genotype, it is also located closest in proximity to the other 16S rRNA Group 4 isolates indicated in green (**Fig 4**). Despite having eight fewer polymorphisms sites for typing, our 16S rRNA gene-based method did exhibit some resolution within the predicted genomic group 4 and could accurately place strains into their more discriminatory MST genotypes. For example, *C*. *burnetii* HHV-WA1, Resson and Arandale with observed MST genotypes of 1–7, 30 were assigned to 16S rRNA Group 3, whereas MST genotype 8 isolates ES-CA1, HHV-WA2, K, Pricilla and P were assigned to 16S rRNA Group 4. The difference in discriminatory power between these two typing schemes is less than 0.07, with D values of 0.7151 and 0.7837 calculated for the 16S rRNA gene-based method and the MST loci-based method, respectively. The only two strains that were not consistent by group were *C*. *burnetii* Mauriet, which was assigned to the predicted genomic group 2, and Dugway 7D77-80, which formed a single branch close to the initial bifurcation point. While the genomic group prediction for Mauriet was 2, this strain is distinctive in that it was the only one in the Hornstra *et al*. [19] study with observed MST genotypes 11–15, 24, 32–34. The MST loci-based SNP assays performed herein for *C*. *burnetii* Dugway 7D77-78 placed this strain in the predicted genomic group 6, which could not be discriminated by 16S rRNA gene typing. This strain, as well as Dugway 5J108-111, was isolated from rodents in Utah [19] and was not assigned a numerical MST. It is suggested that these strains could comprise a novel genotype which is unlike Dugway 5G61-63, a tick isolate that falls within 16S rRNA Group 1 and was previously shown to belong to Genomic Group 1 [42] in the Hendrix *et al*. study [24].

## Discussion

With a broad range animal reservoir and with its potential misuse as a bioterror weapon, an unambiguous laboratory method that can genotype *C*. *burnetii* and help trace infections back to their source during an outbreak is crucial. Our analysis of the complete 16S rRNA led to the classification of all study strains into six distinct groups and allowed us to infer *C*. *burnetii* phylogeny in concordance with other typing methods. 16S rRNA genes contain nine variable regions (V1-V9) that exhibit substantial sequence diversity that is species-specific and can be useful targets for diagnostic assays. However, it has been demonstrated that no single variable region of the 16S rRNA gene can differentiate among all bacteria [43] and analyzing the entire 16S rRNA gene with the nine variable regions is important for scientific investigations. Variations in intra-genomic copies of the 16S rRNA gene must be taken into account for other bacterial biothreat pathogens [35, 44], but *C*. *burnetii* contains a single gene copy so there is no potential for sequence heterogeneity.

Two previous sequencing studies targeted either the 16S rRNA gene [27] or the internal transcribed 16S-23S rDNA spacer (ITS) region [28] to genotype *C*. *burnetii*. Both studies revealed very high levels of sequence homology, but the methods were not useful for epidemiological, clinical or taxonomic purposes [27, 28]. The limited number of strains included in these studies likely contributed to the restricted usefulness of the typing method. Through analysis of a larger and more diverse strain collection, we demonstrated that 16S rRNA gene sequencing can be used for *C*. *burnetii* genotyping.

To avoid any potential loss of genotyping information between methods, we compared the 16S rRNA gene-based typing results to those defined by restriction enzyme banding patterns in the more historic Hendrix *et al*. study [24]. All newly typed isolates overlapping with those isolates used in Hendrix *et al*. [24] were accurately placed into these previously established genomic groups and the discriminatory power of these two typing methods was comparable. These genomic groups also correlate to previous groupings based on plasmid types [22]. Isolates in Groups 1, 2 and 3 all contain the QpH1 plasmid and QpRS is found in the genomes of Group 4 isolates. Members of Group 5 are plasmidless and Dugway isolates in Group 6 contain the QpDG plasmid [24].

We evaluated the congruity of our results and the discriminatory power of the 16S rRNA SNP-based genotyping method by comparing our inference of *C*. *burnetii* phylogeny to phylogeny predicted by the Hornstra *et al*. [19] MST SNP-based approach. Moreover, to expand the dataset for comparison and to build on our existing understanding of phylogeny, we performed MST genotyping for 16 *C*. *burnetii* isolates that have not been previously described. This is of particular importance in order to facilitate inter-laboratory comparisons as *C*. *burnetii* collections are often small due to the culture and containment requirements. While the calculated D values show that 14 SNPs have more discriminatory power compared to the six sites identified among the 16S rRNA gene sequences, a small number of SNPs is suitable for rooting phylogenies if there is a low substitution rate and resolution surrounding the root of the tree [45]. Indeed, 53 of the 55 newly typed strains were assigned to either the correct genomic group as predicted by Hornstra *et al*. [19] or located on the nearest branch indicating the closest evolutionary relationship. We observed instances of both increased and decreased resolution when comparing the genotyping schemes. Using the 16S rRNA SNP-based approach, we could discern different MST genotypes within the predicted genomic group 4, but could not resolve predicted genomic group 1 from its closest neighbor on the tree, genomic group 3. Along with typeability, reproducibility, and ease of interpretation, discriminatory power is an important criteria in evaluating bacterial genotyping methods [46]; however, higher resolution does not necessarily correspond to a more accurate inference of epidemiological relatedness and may be less suitable during analysis of many isolates over an extended period of time [47]. Thus, the genotyping method that is chosen for a particular investigation depends on the epidemiological question being addressed and whether the situation pertains to a localized outbreak or to a more large-scale study [47].

One incongruent result between the two typing approaches was observed for the French isolate *C*. *burnetii* Mauriet. While the genomic group prediction for Mauriet was 2, this strain is unique in that it was the only one in that group with observed MST genotypes 11–15, 24, 32–34 [19]. The 16S rRNA gene-based SNP method places the Mauriet isolate in a separate but neighboring group from all the other predicted genomic group 2 strains which were assigned different MST genotypes. As Mauriet was the only strain in our collection that contained an insertion polymorphism, we compared its 16S rRNA sequence with those available in GenBank. *C*. *burnetii* Z3055, a strain clonal to the one responsible for the large Q fever outbreak in the Netherlands in 2007, also contained this same insertion. *C*. *burnetii* Z3055 was isolated in 1992 from an ewe placenta in Germany and has the same MST 33 genotype and VNTR profile as the strain responsible for the outbreak in the Netherlands [48]. It is possible that the French isolate Mauriet is also related to the Dutch epidemic strain as the outbreak most likely spread from Germany to the Netherlands via France [48].

While no laboratory genotyping method is considered the gold standard for *C*. *burnetii* [21], the 16S rRNA SNP-based method shows promise for a rapid and unambiguous solution that can be used to type strains into previously established genomic groups. Assays targeting these SNPs only require a real-time PCR system and can be completed with a short turnaround time; approximately 2 hours post- DNA extraction [49]. In addition, sequence-based methods are less likely to suffer the complications of inter-and intra-laboratory reproducibility [21]. The proliferation of affordable next generation sequencing platforms has contributed to a marked increase in microbial sequence data including metagenomic studies focused on full length 16S rRNA genes [50]. Unlike SNPs located in the genome-wide MST loci, the 16S rRNA SNPs identified in this study are localized to a 1500 nt region of the *C*. *burnetii* genome. 16S rRNA sequence data collected during metagenomic analyses of clinical or environmental samples could be useful for both identification and now genotyping of *C*. *burnetii* strains. In addition, 16S rRNA SNP genotyping assays could be directly applicable to these samples, in which fragmented DNA may be present, because only small regions require amplification [49, 51]. Also, due to the very low mutation rate of 10^−9^ to 10^−11^ per base pair per generation reported for intracellular bacteria [21], the 16S rRNA SNP targets are considered stable. Future work could comprise development of timely and inexpensive assays for detection of the six 16S rRNA gene-based SNPs including; TaqMan minor-groove binding dual-probe and melt-MAMA assays or by pyrosequencing [19, 52]. SNP-based genotyping could be employed as the gold-standard method for *C*. *burnetii*, but until the field comes to an agreement, harmonization across promising new techniques is crucial.

## Disclaimer

The findings and conclusions in this report are those of the authors and do not necessarily represent the views of the Centers for Disease Control and Prevention or the U.S. Department of Homeland Security. The use of trade names and commercial sources is for identification purposes only and does not imply endorsement by the U.S. Public Health Services, the Department of Health and Human Services, or the U.S. Department of Homeland Security. HPM, BC, JRH, RAP, AC, AC, DH, PPS, LMW, GJK and DS declare no competing financial interests.

## Supporting information

S1 Table SNP signatures among MST loci for *C*. *burnetii* strains. Signatures for these strains, not previously included in the Hornstra *et al*. [19] study, were examined by Melt-MAMA or dual-probe assays.
(DOCX)

## References

[1] Tissot-Dupont H, Amadei MA, Nezri M, Raoult D. Wind in November, Q fever in December. Emerg Infect Dis. 2004;10(7):1264–9. doi: 10.3201/eid1007.030724 15324547 10.3201/eid1007.030724 PMC3323349

[2] Fishbein DB, Raoult D. A cluster of Coxiella burnetii infections associated with exposure to vaccinated goats and their unpasteurized dairy products. Am J Trop Med Hyg. 1992;47(1):35–40. 1636881 10.4269/ajtmh.1992.47.35

[3] Oliphant JW, Gordon DA, et al Q fever in laundry workers, presumably transmitted from contaminated clothing. Am J Hyg. 1949;49(1):76–82. 18108523 10.1093/oxfordjournals.aje.a119261

[4] Maurin M, Raoult D. Q fever. Clin Microbiol Rev. 1999;12(4):518–53. 10515901 10.1128/cmr.12.4.518 PMC88923

[5] Anderson A, Bijlmer H, Fournier PE, Graves S, Hartzell J, Kersh GJ, et al Diagnosis and management of Q fever—United States, 2013: recommendations from CDC and the Q Fever Working Group. MMWR Recomm Rep. 2013;62(RR-03):1–30. 23535757

[6] Haldane EV, Marrie TJ, Faulkner RS, Lee SH, Cooper JH, MacPherson DD, et al Endocarditis due to Q fever in Nova Scotia: experience with five patients in 1981–1982. J Infect Dis. 1983;148(6):978–85. 6655300 10.1093/infdis/148.6.978

[7] Dupuis G, Peter O, Luthy R, Nicolet J, Peacock M, Burgdorfer W. Serological diagnosis of Q fever endocarditis. Eur Heart J. 1986;7(12):1062–6. 3549313 10.1093/oxfordjournals.eurheartj.a062016

[8] Million M, Walter G, Thuny F, Habib G, Raoult D. Evolution from acute Q fever to endocarditis is associated with underlying valvulopathy and age and can be prevented by prolonged antibiotic treatment. Clin Infect Dis. 2013;57(6):836–44. doi: 10.1093/cid/cit419 23794723 10.1093/cid/cit419

[9] Botelho-Nevers E, Fournier PE, Richet H, Fenollar F, Lepidi H, Foucault C, et al Coxiella burnetii infection of aortic aneurysms or vascular grafts: report of 30 new cases and evaluation of outcome. Eur J Clin Microbiol Infect Dis. 2007;26(9):635–40. doi: 10.1007/s10096-007-0357-6 17629755 10.1007/s10096-007-0357-6

[10] Angelakis E, Edouard S, Lafranchi MA, Pham T, Lafforgue P, Raoult D. Emergence of Q fever arthritis in France. J Clin Microbiol. 2014;52(4):1064–7. doi: 10.1128/JCM.03371-13 24430457 10.1128/JCM.03371-13 PMC3993513

[11] Madariaga MG, Rezai K, Trenholme GM, Weinstein RA. Q fever: a biological weapon in your backyard. Lancet Infect Dis. 2003;3(11):709–21. 14592601 10.1016/s1473-3099(03)00804-1

[12] Regis E. The Biology of Doom: The History of America's Secret Germ Warfare Project: Henry Holt and Associates New York; 1999.

[13] Hawker JI, Ayres JG, Blair I, Evans MR, Smith DL, Smith EG, et al A large outbreak of Q fever in the West Midlands: windborne spread into a metropolitan area? Commun Dis Public Health. 1998;1(3):180–7. 9782633

[14] Seshadri R, Samuel J. Genome analysis of Coxiella burnetii species: insights into pathogenesis and evolution and implications for biodefense. Ann N Y Acad Sci. 2005;1063:442–50. doi: 10.1196/annals.1355.063 16481558 10.1196/annals.1355.063

[15] Spicer AJ, Crowther RW, Vella EE, Bengtsson E, Miles R, Pitzolis G. Q fever and animal abortion in Cyprus. Trans R Soc Trop Med Hyg. 1977;71(1):16–20. 558666 10.1016/0035-9203(77)90198-5

[16] Hartzell JD, Peng SW, Wood-Morris RN, Sarmiento DM, Collen JF, Robben PM, et al Atypical Q fever in US soldiers. Emerg Infect Dis. 2007;13(8):1247–9. doi: 10.3201/eid1308.070218 17953104 10.3201/eid1308.070218 PMC2828091

[17] Eldin C, Mahamat A, Demar M, Abboud P, Djossou F, Raoult D. Q fever in French Guiana. Am J Trop Med Hyg. 2014;91(4):771–6. doi: 10.4269/ajtmh.14-0282 25092817 10.4269/ajtmh.14-0282 PMC4183403

[18] Delsing CE, Kullberg BJ, Bleeker-Rovers CP. Q fever in the Netherlands from 2007 to 2010. Neth J Med. 2010;68(12):382–7. 21209463

[19] Hornstra HM, Priestley RA, Georgia SM, Kachur S, Birdsell DN, Hilsabeck R, et al Rapid typing of Coxiella burnetii. PloS one. 2011;6(11):e26201 doi: 10.1371/journal.pone.0026201 22073151 10.1371/journal.pone.0026201 PMC3206805

[20] D'Amato F, Eldin C, Raoult D. The contribution of genomics to the study of Q fever. Future Microbiol. 2016;11(2):253–72. doi: 10.2217/fmb.15.137 26854360 10.2217/fmb.15.137

[21] Toman R, Heinzen R.A., Samuel J.E., Mege J.L. (Eds). Chapter 19. Molecular Typing of Coxiella burnetii (Q Fever) Coxiella burnetii: Recent Advances and New Perspectives in Research of the Q fever Bacterium 2012 p. 381–96.

[22] Samuel JE, Frazier ME, Mallavia LP. Correlation of plasmid type and disease caused by Coxiella burnetii. Infect Immun. 1985;49(3):775–9. 4030104 10.1128/iai.49.3.775-779.1985 PMC261272

[23] Jager C, Willems H, Thiele D, Baljer G. Molecular characterization of Coxiella burnetii isolates. Epidemiol Infect. 1998;120(2):157–64. 9593485 10.1017/s0950268897008510 PMC2809385

[24] Hendrix LR, Samuel JE, Mallavia LP. Differentiation of Coxiella burnetii isolates by analysis of restriction-endonuclease-digested DNA separated by SDS-PAGE. J Gen Microbiol. 1991;137(2):269–76. doi: 10.1099/00221287-137-2-269 1673152 10.1099/00221287-137-2-269

[25] Sekeyova Z, Roux V, Raoult D. Intraspecies diversity of Coxiella burnetii as revealed by com1 and mucZ sequence comparison. FEMS Microbiol Lett. 1999;180(1):61–7. 10547445 10.1111/j.1574-6968.1999.tb08778.x

[26] Nguyen SV, Hirai K. Differentiation of Coxiella burnetii isolates by sequence determination and PCR-restriction fragment length polymorphism analysis of isocitrate dehydrogenase gene. FEMS Microbiol Lett. 1999;180(2):249–54. 10556719 10.1111/j.1574-6968.1999.tb08803.x

[27] Stein A, Saunders NA, Taylor AG, Raoult D. Phylogenic homogeneity of Coxiella burnetii strains as determinated by 16S ribosomal RNA sequencing. FEMS Microbiol Lett. 1993;113(3):339–44. 7505761 10.1111/j.1574-6968.1993.tb06537.x

[28] Stein A, Kruszewska D, Gouvernet J, Raoult D. Study of the 16S-23S ribosomal DNA internal spacer of Coxiella burnetii. Eur J Epidemiol. 1997;13(4):471–5. 9258555 10.1023/a:1007389315808

[29] Glazunova O, Roux V, Freylikman O, Sekeyova Z, Fournous G, Tyczka J, et al Coxiella burnetii genotyping. Emerg Infect Dis. 2005;11(8):1211–7. doi: 10.3201/eid1108.041354 16102309 10.3201/eid1108.041354 PMC3320512

[30] Arricau-Bouvery N, Hauck Y, Bejaoui A, Frangoulidis D, Bodier CC, Souriau A, et al Molecular characterization of Coxiella burnetii isolates by infrequent restriction site-PCR and MLVA typing. BMC Microbiol. 2006;6:38 doi: 10.1186/1471-2180-6-38 16640773 10.1186/1471-2180-6-38 PMC1488860

[31] Weisburg WG, Dobson ME, Samuel JE, Dasch GA, Mallavia LP, Baca O, et al Phylogenetic diversity of the Rickettsiae. J Bacteriol. 1989;171(8):4202–6. 2753854 10.1128/jb.171.8.4202-4206.1989 PMC210191

[32] Masuzawa T, Sawaki K, Nagaoka H, Akiyama M, Hirai K, Yanagihara Y. Identification of rickettsiae isolated in Japan as Coxiella burnetii by 16S rRNA sequencing. Int J Syst Bacteriol. 1997;47(3):883–4. doi: 10.1099/00207713-47-3-883 9226923 10.1099/00207713-47-3-883

[33] Mollet C, Drancourt M, Raoult D. Determination of Coxiella burnetii rpoB sequence and its use for phylogenetic analysis. Gene. 1998;207(1):97–103. 9511749 10.1016/s0378-1119(97)00618-5

[34] Baker GC, Smith JJ, Cowan DA. Review and re-analysis of domain-specific 16S primers. J Microbiol Methods. 2003;55(3):541–55. 14607398 10.1016/j.mimet.2003.08.009

[35] Hakovirta JR, Prezioso S, Hodge D, Pillai SP, Weigel LM. Identification and Analysis of Informative Single Nucleotide Polymorphisms in 16S rRNA Gene Sequences of the Bacillus cereus Group. J Clin Microbiol. 2016;54(11):2749–56. doi: 10.1128/JCM.01267-16 27582514 10.1128/JCM.01267-16 PMC5078553

[36] Altschul SF, Gish W, Miller W, Myers EW, Lipman DJ. Basic local alignment search tool. J Mol Biol. 1990;215(3):403–10. doi: 10.1016/S0022-2836(05)80360-2 2231712 10.1016/S0022-2836(05)80360-2

[37] Hunter PR, Gaston MA. Numerical index of the discriminatory ability of typing systems: an application of Simpson's index of diversity. J Clin Microbiol. 1988;26(11):2465–6. 3069867 10.1128/jcm.26.11.2465-2466.1988 PMC266921

[38] Vogler AJ, Birdsell D, Price LB, Bowers JR, Beckstrom-Sternberg SM, Auerbach RK, et al Phylogeography of Francisella tularensis: global expansion of a highly fit clone. J Bacteriol. 2009;191(8):2474–84. doi: 10.1128/JB.01786-08 19251856 10.1128/JB.01786-08 PMC2668398

[39] Easterday WR, Van Ert MN, Zanecki S, Keim P. Specific detection of bacillus anthracis using a TaqMan mismatch amplification mutation assay. Biotechniques. 2005;38(5):731–5. 15945372 10.2144/05385ST03

[40] Van de Peer Y, Chapelle S, De Wachter R. A quantitative map of nucleotide substitution rates in bacterial rRNA. Nucleic Acids Res. 1996;24(17):3381–91. 8811093 10.1093/nar/24.17.3381 PMC146102

[41] Walter MC, Vincent GA, Stenos J, Graves S, Frangoulidis D. Genome Sequence of Coxiella burnetii Strain AuQ01 (Arandale) from an Australian Patient with Acute Q Fever. Genome Announc. 2014;2(5). 10.1128/genomeA.00964-14 PMC4183872 25278528

[42] Beare PA, Samuel JE, Howe D, Virtaneva K, Porcella SF, Heinzen RA. Genetic diversity of the Q fever agent, Coxiella burnetii, assessed by microarray-based whole-genome comparisons. J Bacteriol. 2006;188(7):2309–24. doi: 10.1128/JB.188.7.2309-2324.2006 16547017 10.1128/JB.188.7.2309-2324.2006 PMC1428397

[43] Chakravorty S, Helb D, Burday M, Connell N, Alland D. A detailed analysis of 16S ribosomal RNA gene segments for the diagnosis of pathogenic bacteria. J Microbiol Methods. 2007;69(2):330–9. doi: 10.1016/j.mimet.2007.02.005 17391789 10.1016/j.mimet.2007.02.005 PMC2562909

[44] Hao H, Liang J, Duan R, Chen Y, Liu C, Xiao Y, et al Yersinia spp. Identification Using Copy Diversity in the Chromosomal 16S rRNA Gene Sequence. PloS one. 2016;11(1):e0147639 doi: 10.1371/journal.pone.0147639 26808495 10.1371/journal.pone.0147639 PMC4726496

[45] Pearson T, Hornstra HM, Sahl JW, Schaack S, Schupp JM, Beckstrom-Sternberg SM, et al When outgroups fail; phylogenomics of rooting the emerging pathogen, Coxiella burnetii. Syst Biol. 2013;62(5):752–62. doi: 10.1093/sysbio/syt038 23736103 10.1093/sysbio/syt038 PMC3739886

[46] Lipuma JJ. Molecular tools for epidemiologic study of infectious diseases. Pediatr Infect Dis J. 1998;17(8):667–75. 9726338 10.1097/00006454-199808000-00002

[47] Coenye T, Spilker T, Martin A, LiPuma JJ. Comparative assessment of genotyping methods for epidemiologic study of Burkholderia cepacia genomovar III. J Clin Microbiol. 2002;40(9):3300–7. doi: 10.1128/JCM.40.9.3300-3307.2002 12202570 10.1128/JCM.40.9.3300-3307.2002 PMC130787

[48] D'Amato F, Rouli L, Edouard S, Tyczka J, Million M, Robert C, et al The genome of Coxiella burnetii Z3055, a clone linked to the Netherlands Q fever outbreaks, provides evidence for the role of drift in the emergence of epidemic clones. Comp Immunol Microbiol Infect Dis. 2014;37(5–6):281–8. doi: 10.1016/j.cimid.2014.08.003 25249233 10.1016/j.cimid.2014.08.003

[49] Huijsmans CJ, Schellekens JJ, Wever PC, Toman R, Savelkoul PH, Janse I, et al Single-nucleotide-polymorphism genotyping of Coxiella burnetii during a Q fever outbreak in The Netherlands. Appl Environ Microbiol. 2011;77(6):2051–7. doi: 10.1128/AEM.02293-10 21257816 10.1128/AEM.02293-10 PMC3067327

[50] Shin J, Lee S, Go MJ, Lee SY, Kim SC, Lee CH, et al Analysis of the mouse gut microbiome using full-length 16S rRNA amplicon sequencing. Sci Rep. 2016;6:29681 doi: 10.1038/srep29681 27411898 10.1038/srep29681 PMC4944186

[51] Tilburg JJ, Melchers WJ, Pettersson AM, Rossen JW, Hermans MH, van Hannen EJ, et al Interlaboratory evaluation of different extraction and real-time PCR methods for detection of Coxiella burnetii DNA in serum. J Clin Microbiol. 2010;48(11):3923–7. doi: 10.1128/JCM.01006-10 20826645 10.1128/JCM.01006-10 PMC3020840

[52] Amoako KK, Thomas MC, Janzen TW, Goji N. Rapid SNP Detection and Genotyping of Bacterial Pathogens by Pyrosequencing. Methods Mol Biol. 2017;1492:203–20. doi: 10.1007/978-1-4939-6442-0_15 27822867 10.1007/978-1-4939-6442-0_15

