# Supplementary material for: Phylogenetic inference of Coxiella burnetii by 16S rRNA gene sequencing
Source: PLoS One. 2017 Dec 29;12(12):e0189910. doi: 10.1371/journal.pone.0189910 (PMC5747434; doi:10.1371/journal.pone.0189910)
Supplement: S1 Table — Signatures for these strains, not previously included in the Hornstra et al. [19] study, were examined by Melt-MAMA or dual-probe assays. (DOCX) [file pone.0189910.s001.docx]

**S1 Table.**  SNP signatures among MST loci for *C. burnetii* strains.

Cox18 bp166

Cox20 bp155

Cox51 bp67

Cox37 bp215

Cox56 bp10

Cox57 bp327

Cox51 bp492

Cox22 bp 118

Cox5 bp109

Cox18 bp34

Cox51 bp356

Cox22 bp91

Cox18 bp376

Cox5 bp81

| D. occidentales | G | T | A | T | T | T | C | G | G | T | G | T | G | G |
| --- | --- | --- | --- | --- | --- | --- | --- | --- | --- | --- | --- | --- | --- | --- |
| A. americanum | G | T | A | T | T | T | C | G | G | T | G | T | G | G |
| Dugway 7D 77-80 | G | C | G | G | T | T | C | G | A | T | G | T | A | G |
| El Tayeb | G | T | A | T | T | T | C | G | G | T | G | T | G | G |
| O. mengnini | G | T | A | T | T | T | C | G | G | T | G | T | G | G |
| RSA 335 | G | T | A | T | T | T | C | G | G | T | G | T | G | G |
| R. sanguineus | G | T | A | T | T | T | C | G | G | T | G | T | G | G |
| l. scapularis | G | T | A | T | T | T | C | G | G | T | G | T | G | G |
| ES-MT1 | G | C | G | G | C | T | A | G | A | T | G | C | G | G |
| GP-MT2 | G | C | G | G | C | T | A | G | A | T | G | C | G | G |
| GP-WA1 | G | C | G | G | C | T | A | G | A | T | G | C | G | G |
| GS-MT1 | G | C | G | G | C | T | A | G | A | T | G | C | G | G |
| ES-WA1 | G | C | G | G | C | T | A | G | A | T | G | C | G | G |
| ES-WA2 | G | C | G | G | C | T | A | G | A | T | G | C | G | G |
| GP-AF1 | G | C | G | G | C | T | A | G | A | T | G | T | G | G |
| Q218 | G | C | G | G | C | T | A | G | A | T | G | C | G | G |
